# A comprehensive review on nutritional, nutraceutical, and industrial perspectives of perilla (*Perilla frutscens* L.) seeds – An orphan oilseed crop

**DOI:** 10.1016/j.heliyon.2024.e33281

**Published:** 2024-06-18

**Authors:** Simardeep Kaur, Karishma Seem, Ansheef Ali, Sandeep Jaiswal, Praveen Gumachanamardi, Gurkanwal Kaur, Naseeb Singh, Letngam Touthang, Sanjeev Kumar Singh, Rakesh Bhardwaj, Binay K. Singh, Vinay Kumar Mishra, Amritbir Riar

**Affiliations:** a ICAR-Research Complex for North Eastern Hill Region, Umiam, Meghalaya, 793103, India; b ICAR-Indian Agricultural Research Institute, New Delhi, 110012, India; c ICAR-Indian Agricultural Research Institute, Assam, 734301, India; d Punjab Agricultural University, Ludhiana, Punjab, 141004, India; e Krishi Vigyan Kendra, Phek, Nagaland, 797107, India; f ICAR-National Bureau of Plant Genetic Resources, New Delhi, 110012, India; g Department of International Cooperation, Research Institute of Organic Agriculture FiBL, Frick, Switzerland

**Keywords:** Perilla, Phytochemicals, Biological activities, Industrial applications, Blending

## Abstract

There is a growing need to mainstream orphan or underutilized crops to enhance nutritional security and sustainable agriculture. Among these, *Perilla frutescens* L. is an important crop due to its rich nutritional and phytochemical content which makes it significant in nutrition, medicine, and industrial sector. Perilla seeds are mainly rich in ω-3 fatty acids, dietary fiber, amino acids, vitamins, and minerals, high α-linolenic acid, which contributes to their health benefits. This review explores the nutritional profile of perilla seeds and highlights its unique composition compared to other oilseed crops. It also analyzes the phytochemical components of perilla seeds and their various biological activities, including antioxidant, antidiabetic, antiobesity, cardioprotective, anticancer, antimicrobial, neuroprotective, and anti-inflammatory effects. These activities demonstrate the potential of perilla seeds in both pharmaceutical and food sectors. The review also covers recent advancements in genomics and transgenic research discussing potential areas for crop improvement. Additionally, it explores the use of perilla seeds in functional foods, blending perilla oil with other oils, and their applications in enhancing product formulations. This review offers valuable insights for researchers, students, policymakers, environmentalists, and industry professionals by detailing the potential of perilla seeds across various sectors. The findings support sustainable agriculture, crop diversification, and innovative product development, thus contributing to the integration of perilla into mainstream agriculture.

## Highlights

• Comprehensive analysis of nutritional and phytochemical profile of Perilla seeds.
• Exploration of antioxidant, antidiabetic, and anti-inflammatory properties etc.
• Discussion on advancements in genomics for enhancing perilla seed oil content.
• Innovative applications in functional foods and implications for industries.
• Future directions in augmenting Perilla in mainstream agriculture.

## 1 Introduction

Orphan crops, also known as underutilized or neglected crops, refer to agricultural produce lacking widespread global recognition or extensive cultivation. Mainstreaming orphan crops is vital for enhancing food and nutritional security due to their high nutritional value and resilience to diverse environmental conditions. By diversifying agricultural practices, these crops contribute to ecological and environmental security through reduced monoculture pressure, improved soil health, and conservation of biodiversity [[1], [2], [3]]. These crops represent abundant sources of nutrients, biofuels, medicinal compounds, and industrial raw materials. They hold significant potential for sustainable development in low-income nations and appeal to western consumers seeking healthier food options [1].

*Perilla frutescens* (L.) Britton (2n = 40), commonly known as perilla, is an orphan oilseed crop native to East Asia, particularly China, Korea, Japan, India, and Thailand. Belonging to the family Lamiaceae, subfamily Lamioideae, tribe Saturejeae, and subtribe Perillinae, this annual herbaceous crop bears small self-pollinating flowers and brown fruits containing black to white or various shades of grey or brown seeds [[4], [5], [6]]. The seeds contain an oil content ranging from 30 % to 45 %. Perilla is represented by two primary varieties: *P. frutescens* var. Frutescens, predominantly utilized for its seeds and oil, and P*. frutescens* var. Crispa, utilized in traditional medicine applications. Cultivated P*. frutescens* var. Frutescens is tetraploid, whereas wild Perilla species typically exhibit a diploid genetic makeup [7].

Globally, perilla represents an opportunity to address nutritional deficiencies due to its diverse nutritional profile. Its seeds are rich in essential fatty acids and various bioactive compounds including polyphenols. Perilla seed oil (PSO), with approximately 60 % ω-3 α-linolenic acid, ranks high among vegetable oils [4,8], with over 90 % unsaturated fatty acids, particularly α-linolenic acid, associated with health benefits including reduced serum cholesterol, lowered risk of colon cancer, and prevention of excessive visceral adipose tissue growth [9]. The flavonoids in perilla seeds offer additional health benefits, including anti-diabetic, antioxidant, anticancer, and anti-inflammatory properties [[10], [11], [12]].

Beyond its nutritional and nutraceutical values, perilla has wider applications across various industries. For instance, perilla oil is widely used in cosmetics to enhance skin health, and pharmaceutical companies are exploring its medicinal properties for drug development and dietary supplements. In the functional foods sector, perilla-derived products show significance in improving overall health and well-being. The present study provides a comprehensive overview of research advancements in the nutritional, nutraceutical, and industrial applications of perilla while highlighting its potential as an alternative to synthetic food supplements and chemical drugs. Furthermore, it discusses biotechnology-led crop improvement initiatives for perilla. The key findings of this review article holds immense importance in addressing the critical aspects of sustainable, cost-effective food production and enhancing nutritional security. By exploring multiple roles of perilla seeds, it offers a pathway towards sustainable nutrition and economic viability. Readers can gain valuable insights to navigate towards a more resilient and diversified food system, enhancing both personal and global nutritional security.

## 2 Methodology

**Fig. 1:**
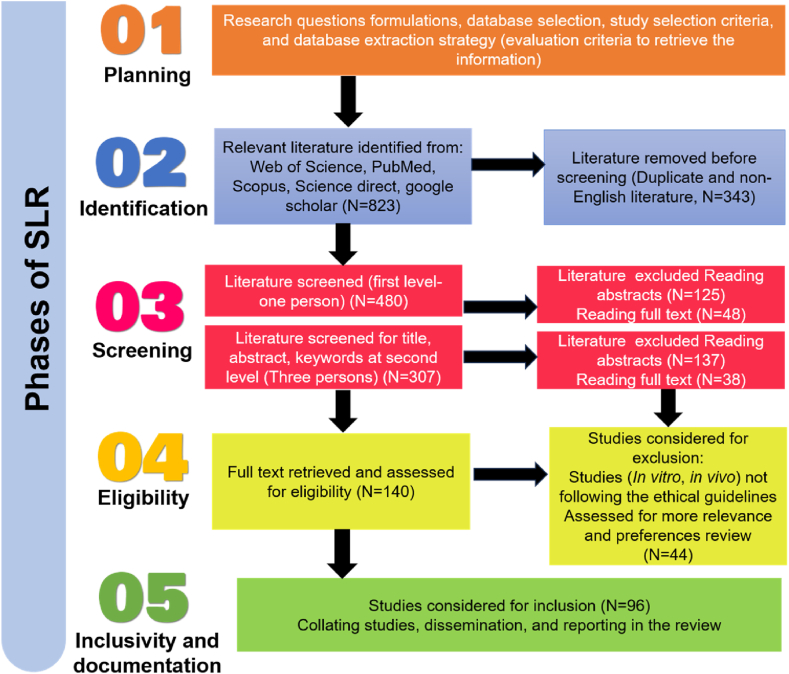
Illustartion of systematic literature review (SLR) for the present investigation.

In the present investigation, a comprehensive exploration of perilla, covering its myriad uses, nutritional composition, phytochemistry, pharmacological properties, industrial potential, and biotechnology interventions up to the year 2023, was assembled and key databases such as Web of Science, Scopus, Google Scholar, PubMed, and ScienceDirect were chosen to ensure a thorough exploration of relevant studies. The initial identification phase involved sifting through hundreds of potential studies with a focus on eliminating duplicates and non-English publications. The screening process employed a careful examination of titles, abstracts, and keywords against predefined inclusion/exclusion criteria, resulting in the exclusion of studies deemed irrelevant or unsuitable for the present review (Fig. 1). Moving to the eligibility phase, full texts of the remaining studies were subjected to a rigorous assessment by 3–4 different individuals to ensure adherence to ethical guidelines and alignment with the established research question, further refining the selection. Finally, the synthesis and reporting was done by drawing insights from the remaining pool of high-quality studies (N = 96) identified through this systematic process. The findings from these selected studies form the robust foundation of the review and their details are summarized and interpreted in the current review, providing a comprehensive and insightful overview of the current state of knowledge on the research topic. The scrupulous curation and synthesis of data from diverse sources enhance the significance of this review, imparting invaluable insights for researchers and professionals within the domain of crop science.

## 3 Nutritional composition of perilla seeds

### 3.1 Lipids/fatty acid profile

Perilla seed oil contains a total lipid content of approximately 40 %, where neutral lipids constitute 91.2–93.9 %, glycolipids range from 3.9 to 5.8 %, and phospholipids contribute around 2–3%. Within neutral lipids, triacylglycerol accounts for 88.1–90 %, sterol ester for 4.1–6.2 %, and hydrocarbons for 1.9–2.7 %. Important saturated fatty acids found in PSO include lauric acid, myristic acid, pentadecanoic acid, palmitic acid, heptadecanoic acid, stearic acid, arachidic acid, and behenic acid, comprising a total of 7–9% of the oil content [13]. The unsaturated fatty acids include monounsaturated fatty acids (MUFAs) such as palmitoleic acid (16:1 *cis*-7), oleic acid (18:1 *cis*-9), eicosenoic acid (20:1 *cis*-11), and polyunsaturated fatty acids (PUFAs) including linoleic acid (18:2 *cis*-9,12), α-linolenic acid (18:3 *cis*-9,12,15), eicosadienoic acid (20:2 *cis*-11,14), and eicosatrienoic acid (20:3 *cis*-11,14,17) [14]. Remarkably, PSO is known for its high levels of unsaturated fatty acids, mainly linolenic acid, oleic acid, and linoleic acid, ranging from 51.2 % to 64 %, 11.9 %–23.8 %, and 12.2 %–18.6 %, respectively [13], surpassing several conventional oil sources such as linseed, sesame, mustard, soy, and sunflower [15,16] (Table 1).

**Table 1:** Fatty acid composition of perilla seed oil vis-à-vis other edible oils.

Vegetable oils	Fatty acids %			Essential fatty acids %		Ratio	
	SFA	MUFA	PUFA	ω-6	ω-3	ω-6/ω-3	SFA:MUFA:PUFA
**Perilla oil**	9	14	78	13	66	0.2	1:1.6:8.7
**Mustard oil**	3	68	29	15	14	1	1:22.6:9.6
**Soybean oil**	15	24	61	54	7	8	1:1.6:4.1
**Groundnut oil**	22	45	33	32	1	32	1:2:1.5
**Sunflower oil**	12	19	69	68	1	68	1:1.6:5.8
**Sesame oil**	19	42	39	42	0.5	84	1:2.2:2.1
**Safflower oil**	9	13	78	78	0	–	1:1.4:8.6
**Linseed oil**	10	21	69	16	53	2	1:2.1:6.9

The ω-6 to ω-3 fatty acid ratio in PSO is considerably low at 0.20 [4,18], significantly below the recommended optimal range of 5–10 for human health (WHO Interim Summary, 2008). Incorporating perilla oil into culinary practices, whether by direct addition to food or as a substitute for hydrogenated oil or cream in baking, offers a means to contribute to rebalancing the ω-6/ω-3 ratio in the diet [19]. PSO's exceptional ω-3 content at 66 % surpasses that of all other oils, making it a preferred choice for those prioritizing essential fatty acids for potential cardiovascular and anti-inflammatory benefits. Furthermore, its low saturated fat content (9 %) and favourable polyunsaturated to monounsaturated fat ratio position it as a good option for a health-conscious diet. For further insights, Table 1 presents a comparison of the fatty acid composition of PSO compared with other edible oils.

### 3.2 Protein and amino acid profile

Perilla seeds contain high protein content, ranging from 15.7 % to 23.9 %, primarily concentrated in the seed kernel [9,20]. When compared to conventional oil sources like mustard (20.0 %), cotton (19.4 %), linseed (20.3 %), sunflower (19.8 %), coconut (23.9 %), and almond (20.8 %), perilla seeds exhibit superior protein quality and higher content. Longvah et al. reported that essential amino acids constitute 39 % of the total amino acid content in perilla seeds, with lysine being limited [9]. Table 2 delineates the amino acid composition of perilla seed meal (PSM) in comparison to seed meals of other edible oils. Interestingly, hulling enhances the protein content, with the hull itself containing 5 % protein [14,20].

**Table 2:** Amino acid composition (g/100 g of protein) in seed meals of perilla vis-à-vis other edible oil sources.

Crop	Perilla	Mustard	Soybean	Groundnut	Sunflower	Sesame	Safflower	Linseed
**Alanine**	3.87	5.71	4.20	2.78	4.59	2.83	2.6	4.4
**Arginine**	10.81	6.82	8.00	5.62	10.12	7.45	5.28	9.2
**Aspartic acid**	9.88	7.49	12.10	9.71	10.02	9.88	3.23	9.3
**Cysteine**	2.70	0.43	1.40	1.65	0.31	0.15	–	1.1
**Glutamic acid**	15.84	19.18	20.40	7.15	22.45	16.54	8.82	–
**Glycine**	4.88	5.51	4.20	2.96	5.35	2.06	1.12	5.8
**Histidine**	2.32	3.87	2.70	2.82	2.79	2.25	1.22	2.2
**Isoleucine**	4.86	3.75	4.30	4.77	4.40	4.85	1.41	4.0
**Leucine**	8.31	8.11	7.80	7.03	6.92	7.57	3.92	5.8
**Lysine**	3.12	6.51	6.50	5.25	3.87	5.06	1.36	4.0
**Methionine**	3.17	2.21	1.40	2.34	2.26	1.87	0.47	1.5
**Phenylalanine**	4.18	5.61	5.40	3.79	5.32	6.34	3.15	4.6
**Proline**	3.30	4.60	5.30	2.12	3.61	4.08	2.95	3.5
**Serine**	4.00	5.40	5.70	5.04	4.05	6.62	1.61	4.5
**Threonine**	3.03	5.81	3.60	3.12	3.70	4.85	1.03	3.6
**Tryptophan**	–	1.47	1.00	7.12	1.57	1.25	0.22	1.8
**Tyrosine**	3.09	5.09	4.10	3.87	3.21	6.61	1.82	2.3
**Valine**	5.65	4.45	4.50	5.08	5.29	5.44	2.47	4.6

Hydrolyzed and fractionated PSM protein exhibits potent antioxidant properties, attributed to its low molecular weight (<3 kDa) and specific amino acids such as leucine, isoleucine, proline, and serine. Ground perilla cake emerges as another valuable source, containing 31.54 % crude protein and essential amino acids (138.34 mg/g), primarily leucine (28.87 mg/g), lysine (19.52 mg/g), and methionine (10.94 mg/g). Studies also suggest that perilla seed by-products, including meal and cake, can be utilized as natural antioxidants and incorporated into animal diets to enhance production and health [[27], [28], [29]].

### 3.3 Vitamin and mineral composition of perilla seeds

Perilla seeds exhibit a distinctive vitamin composition characterized by lower levels of thiamin, riboflavin, niacin, biotin, and folate; however, they contain a substantial amount of vitamin E, predominantly in the form of γ-tocopherol, constituting over 95 % of the total tocopherol content [19,30]. Vitamin E, known for its antioxidant properties, finds versatile applications in food, oils, livestock feeds for enhanced meat quality, and as a dietary supplement for humans.

Moreover, perilla seeds contain essential minerals such as calcium (Ca), iron (Fe), zinc (Zn), magnesium (Mg), and phosphorus (P) in considerable amounts. This mineral abundance aids the body in metabolizing proteins and carbohydrates. However, copper (Cu) and chromium (Cr) levels in perilla seeds are relatively low compared to other oilseed crops, with concentrations of 0.20 mg/100 g and 17.6 μg/100 g, respectively. It's noteworthy that excessive concentrations of copper (beyond 7.3 mg/100 g) and chromium (beyond 0.03 mg/100 g) are considered toxic (Parveen et al., 2003). Table 3 illustrates the proximate mineral composition of perilla seeds in comparison to other oilseeds.

**Table 3:** Proximate composition of the inorganic nutritional content of perilla seeds in comparison to other oilseeds (all values are per 100 g seed).

Component	Perilla	Mustard	Soybean	Groundnut	Sunflower	Sesame	Safflower	Linseed
**Moisture (g)**	7.4	8.5	7.84	5.5	5.5	–	5.5	6.5
**Protein (g)**	17.4	20.0	36.49	18.3	19.8	23.53	13.5	20.3
**Fat (g)**	51.7	39.7	19.94	43.3	52.1	52.86	25.6	37.1
**Ash (g)**	3.6	4.2	4.9	5.2	3.7	4.75	2.6	2.4
**Carbohydrate (g)**	20.3	23.8	30.16	25	17.9	14.52	17.9	28.9
**Energy (kcal)**	615.0	541	446	563	620	–	346	530
**Phosphorus (mg)**	710	700	704	570	670	–	823	370
**Magnesium (mg)**	275	–	280	–	118	52.53	–	–
**Calcium (mg)**	269	490	277	1450	64	45.33	236	170
**Iron (mg)**	9.0	7.9	15.7	9.3	5.6	3.78	4.6	2.7
**Manganese (mg)**	4.8	2.56	2.517	1.32	–	0.23	1.1	–
**Zn (mg)**	4.7	4.8	4.89	12.2	3.78	1.46	5.2	–
**Copper (mg)**	0.18	0.83	–	2.29	1.74	0.73	1.58	–
**Chromium (μg)**	20	63	–	87	–	–	45	–

**Table 4:** Phytochemical profile of perilla seeds (all values are expressed in μg/g seed).

Bioactive compound	Concentration	Reference
Caffeic acid	23.1	[35]
Rosmarinic acid-3-*O*-glucoside	1547.0	
Rosmarinic acid	2757.0	
Luteolin	207.9	
Apigenin	155.7	
Chrysoeriol	83.2	
Policosanols	721	[36]
Caffeic acid	3.0	[37,38]
Luteolin-7-O-glucoside	11.1	
Rosmarinic acid-3-O-glucoside	1592.0	
Rosmarinic acid	1746.5	
Luteolin	19.6	
Apigenin	14.0	
Chrysoeriol	16.5	
Dehydroxyl-rosmarinic acid-3-o-β-D-glucoside	28.13	[39]
Rosmarinic acid-3-o-β-D-glucoside	350.72	
Rosmarinic acid	535.20	
Rosmarinic acid methyl ester	2.76	
Luteolin	28.69	
Luteolin-5-o-glucoside	3.55	
Apigenin	9.88	
Caffeic acid	33.44	
Caffeic acid-3-o-β-D-glucoside	90.31	
Vanillic acid	8.57	
Cimidahurinine	74.91	

## 4 Phytochemical profile of perilla seeds

Perilla, known for its multifaceted health benefits, harbors a plethora of phytochemicals with promising applications against a spectrum of chronic diseases. Irrespective of plant type, importance of the studies on such high value phytochemicals has tremendously increased in the last decade due to its diverse applications. A study conducted by Lee et al. on Korean perilla cultivars unveiled nine major phytochemicals in seeds, with rosmarinic acid-3-O-glucoside and rosmarinic acid emerging as predominant compounds (>95 %) [37]. Subsequent investigations by Zhou et al. reported the antioxidant potential of seed flour, emphasizing the role of rosmarinic acid-3-O-glucoside and rosmarinic acid [39]. Moreover, novel compounds such as 3′-dehydroxyl-rosmarinic acid were identified in perilla seeds. Perilla seeds also contain tocopherols (α-tocopherol: 43.81 ± 0.38, γ-tocopherol: 344.56 ± 0.05, δ-tocopherol: 23.10 ± 1.48), Sterols (Campesterol: 321.16 ± 5.16, Stigmasterol: 177.50 ± 2.65, β-sitosterol: 2773.85 ± 12.04), and Policosanols (C21–OH: 4.51 ± 0.12) [14]. Table 4 exhibits phytochemical composition of perilla from few research studies. Apart from this, phytochemicals extracted from perilla seeds were primarily studied for their anti-disease properties, such as anti-cancerous effect of rosmarinic acid, anti-inflammatory activities, wound healing properties [28,40,35] etc. Still, further studies should be conducted to understand the diversity of phytochemicals in perilla seeds and their role in human nutrition.

## 5 Biological activities of perilla seeds

Perilla seeds are recognized for their diverse biological activities, including antioxidant, antidiabetic, antiobesity, cardioprotective, anticancer, antimicrobial, neuroprotective, and anti-inflammatory properties (Table 5).

**Table 5:** Summary of biological activities of perilla seeds.

Variety	Type of extract	Bioactive compound identified	Model	Major findings	Reference
**Antioxidant**					
*P. frutescens*	Dichlorometane, Ethyl acetate	Rosmarinic acid, caffeic acid, ferulic acid, caffeic acid-3-Oglucoside, and rosmarinic acid-3-O-glucosid	Sprague–Dawley rats	Antioxidant and anti-inflammatory effects	[35,38]
**Anti-diabetic**					
*P*. *frutescens* (L.) Britton var	Ethyl acetate soluble fraction of methanol extract	Chlorogenic acid and rosmarinic acid	Type 2 diabetes mice model	Influence insulin secretion from pancreatic beta cells, modulation of the AMPK pathway and inhibition of gluconeogenesis in the liver	[41,42,43]
**Anti-Obesity**					
*P*. *frutescens*	Cold-pressed *Perilla* oil	α-linolenic acid (ALA)	C57BL/6 N mice	Promoted the removal of cholesterol from hepatocytes (liver cells) by converting it into bile acids and increasing fecal cholesterol excretion, promoting autophagy to remove excess lipids in the liver	[44,45]
**Cardioprotective**					
*P*. *frutescens*	Seed oil	ALA	Male New Zealand white rabbits and Sprague-Dawley rats, Wister rats	Lipid accumulation in the thoracic aorta and liver, delaying arterial occlusion, increased serum level of n-3 fatty acid, reduce both cholesterol and triglyceride levels, modulates neuropeptides that control appetite,	[9,46]
**Anti-cancer**					
*P*. *frutescens*	ethanolic extract	omega-3 polyunsaturated fatty acids	A549 cells MCF7 B16F10 melanoma cells	Antiproliferative activity, reduce the activity of ornithine decarboxylase, collagenase inhibitory effects and an *anti*-melanogenic effect, anti-inflammatory effects	[8,[47], [48], [49]]
**Antimicrobial**					
*P*. *frutescens* Britton var. japonica Hara	Ethyl acetate seed extracts	–	–	combats oral bacteria associated with dental caries (cavities) and periodontal diseases, reduce staphylococcal enterotoxins, TSST-1	[50,51]
**Neuroprotective**					
*P. frutescens*	Alcoholic extract	Luteolin	Rat SH-SY5Y cells (human neuroblastoma cells	Improvements in their learning ability and memory, prevent neurodegenerative disorders, including Alzheimer's disease, Parkinson's disease, and cerebral ischemia disorders. prevent hyperphosphorylation of tau protein.	[52,53]
**Anti-inflammatory**					
*P. frutescens*	Seed oil	ALA, Rosmarinic acid and luteolin	Wistar rats	Anti-tussive (cough-suppressing), expectorant (mucus-clearing), and anti-inflammatory effects, suppresses inflammatory markers	[8,54,55]

### 5.1 Antioxidant activity

Antioxidants play an important role in protecting cells against oxidative stress by neutralizing harmful free radicals in the body. Perilla seeds exhibit potent antioxidant activity attributed to various bioactive compounds, including polyphenols and essential fatty acids. Some of these compounds include, rosmarinic acid, caffeic acid, ferulic acid, caffeic acid-3-O-glucoside, and rosmarinic acid-3-O-glucoside, while major antioxidants within flavonoids include apigenin and luteolin [38,56]. Luteolin, for instance, demonstrates the ability to protect primary neurons from oxidative damage induced by hydrogen peroxide, thus suggesting promise in preventingneurodegenerative diseases linked with oxidative stress [57]. Moreover, the methanol extract (80 %) of perilla seeds shows robust antioxidant activities in commonly used radical assays like 2,2-diphenyl-1-picrylhydrazyl (DPPH) and 2,2′-azino-bis (3-ethylbenzthiazoline-6-sulphonic acid) (ABTS) radicals [37]. Particularly, brown perilla seeds exhibit high antioxidant capacity, surpassing white perilla, flax, and chia seeds [18].

Additionally, peptides derived from perill seed meal demonstrate potent antioxidant activities, attributed to their low molecular weight (<3 kDa) and specific amino acids, including leucine, isoleucine, proline, and serine [28]. Two dipeptides, Tyr-Leu (YL) and Phe-Tyr (FY), identified in defatted perilla seed protein enzymatic hydrolysate exhibit high antioxidant activity, effectively neutralizing harmful free radicals and inhibiting lipid peroxidation in rat liver [58]. These dipeptides hold potential applications in improving food quality and addressing health and cosmetic issues related to oxidative stress and excessive reactive oxygen species (ROS).

### 5.2 Antidiabetic activity

Perilla sprouted seed extract has gained attention for its potential antidiabetic effects. Paek et al. identified chlorogenic acid and rosmarinic acid as aldose reductase enzyme inhibitors in an ethyl acetate-soluble fraction of methanol extract, thus helping to mitigate the biochemical changes that contribute to diabetic complications [41]. Another study carried out on the antidiabetic effect of perilla seed sprouts in a type 2 diabetes mouse model, suggested that the supplementation of these sprouts had several positive effects on metabolic health and glucose regulation in the experimental model. Perilla sprouted seed extract may help improve insulin sensitivity, hyperglycemia, and glucose tolerance. Enhanced insulin sensitivity means that the cells can better respond to insulin, leading to improved glucose uptake and regulation as well as adenosine monophosphate-activated protein kinase (AMPK) activation. Regulating this process can help maintain blood glucose levels within a healthy range [42]. A study also highlighted the potential role of perilla oil supplementation in modulating the gut microbiota with a reduction in potentially problematic bacteria like *Blautia* and an increase in beneficial bacteria like *Lactobacillus*, which could contribute to improved glucose metabolism and its potential relevance to prebiotic/metabolic health [59].

### 5.3 Antiobesity activity

Obesity, a prevalent nutritional disorder globally, is characterized by excessive body fat accumulation, posing significant health risks. PSO serves as a rich source of ω-3 unsaturated fatty acids, notably α-linolenic acid, known for its positive impact on lipid metabolism [44]. Studies involving rats have demonstrated that long-term, high-level dietary cholesterol intake hinders key aspects of cholesterol metabolism in the liver [40]. Administering a perilla oil-rich diet for 16 weeks reduces liver fat and inflammation, addressing hepatic steatosis, or fatty liver disease. This dietary intervention also prevents the elevation of triglyceride and total cholesterol levels by facilitating cholesterol removal from hepatocytes, converting it into bile acids, and increasing faecal cholesterol excretion [60]. This suggests a potential role of PSO in lowering cholesterol levels and mitigating complications associated with high dietary cholesterol intake. Another study, utilizing perilla oil treatment at varying doses, revealed antiobesity effects in a high-fat diet-induced obesity model over 12 weeks, indicating potential for perilla oil as a dietary intervention in obesity management [61]. Scientific evidence in an obesity model suggests that α-linolenic acid from perilla oil may reduce hepatic steatosis by modulating the cellular response to endoplasmic reticulum stress, specifically by promoting autophagy to remove excess lipids in the liver [62].

### 5.4 Cardioprotective activity

Perilla oil as a dietary component, appears to have favourable impacts on lipid metabolism, liver health, and cardiovascular well-being, particularly in the context of a high-fat diet. Research suggests that perilla oil may positively influence lipid metabolism and regulate serum cholesterol levels by reducing the expression of β-hydroxy β-methylglutaryl-CoA (HMG-CoA) reductase, an enzyme involved in cholesterol synthesis [9,40]. Treatment with PSO has been associated with decreased lipid accumulation in the thoracic aorta and liver compared to palm oil treatment, potentially linked to its unique fatty acid composition, including high levels of α-linolenic acid [62]. A study comparing PSO and palm oil effects on lipid metabolism in mice fed a high-fat diet for 90 days, found that mice treated with PSO experienced significant reductions in serum cholesterol levels, hepatic triglyceride levels, and lipid accumulation in the liver and thoracic aorta compared to palm oil treatment [42]. PSO may also contribute to the prevention of excessive blood clot formation, a key factor in cardiovascular events. Incubating rabbit platelets with PSO resulted in reduced platelet aggregation induced by collagen and thrombin, suggesting a favourable impact on cardiovascular health [46].

### 5.5 Anticancer activity

PSO has shown efficacy in delaying breast cancer progression in rats and humans [14]. It effectively reduces the activity of ornithine decarboxylase in the colonic omentum and inhibits breast cancer incidence caused by the chemical carcinogen 7-sulfooxymethyl-12-methylbenz-α-anthracene (SMBA), with moderately good effects observed in clinical trials. Perilla seed oil has also demonstrated effectiveness in inhibiting the growth of breast cancer cells, including MCF7 cells [47,48]. Combining PSO with conventional cancer treatments, such as epirubicin and paclitaxel, in breast cancer patients may yield beneficial effects. Perilla pomace, a by-product of seed processing, exhibits collagenase inhibitory effects and *anti*-melanogenic properties on B16F10 melanoma cells, potentially reducing melanin production associated with hyperpigmentation and uneven skin tone. Importantly, the extract does not induce cytotoxicity [49]. Luteolin-rich fractions from perilla seed meal, at concentrations ranging from 25 to 100 μg/mL, show potential to induce anti-inflammatory effects targeted against inflammation induced by the spike glycoprotein S1 of SARS-CoV-2 in A549 lung cells, commonly used in studying non-small cell lung cancer (Lin et al., 2010), during the incidence of long COVID [8].

### 5.6 Antimicrobial activity

A study by Yamamoto and Ogawa (2002) investigated the antibacterial activity of ethyl acetate seed extracts from *P*. *frutescens* Britton var. *japonica* Hara against oral cariogenic *Streptococci* and the periodontopathic bacterium *Porphyromonas gingivalis*. The findings suggested that ethyl acetate seed extracts, rich in luteolin content, may have potential applications in combating oral bacteria associated with dental caries (cavities) and periodontal diseases [50]. Additionally, perilla seed oil demonstrated a dose-dependent reduction in the production of various toxins in *Staphylococcus aureus*. These toxins, including α-toxin, enterotoxins A and B (major Staphylococcal enterotoxins), and toxic shock syndrome toxin 1 (TSST-1), are virulence factors produced by *S*. *aureus* and contribute to the symptoms and severity of Staphylococcal infections [51].

### 5.7 Neuroprotective activity

Luteolin from perilla seeds shows neuroprotective properties by protecting primary cortical neurons from oxidative stress-induced damage and enhancing neuronal viability [57]. Rats fed a diet containing PSO demonstrate improvements in learning ability and memory. These findings suggest that PSO may hold promise in addressing neurodegenerative disorders like Alzheimer's disease and Parkinson's disease. These conditions are often linked to oxidative stress and neuronal damage and luteolin's antioxidant properties may help protect against such damage by elevating levels of antioxidant enzymes, including catalase (CAT) and glutathione (GSH), in primary cortical neurons. PSO, along with its active constituent α-linolenic acid, has the potential to protect neurons from the toxic effects of β-amyloid peptides, reduce oxidative stress, and prevent hyperphosphorylation of tau protein. Tau protein, primarily found in neurons, stabilizes microtubules which are crucial for the cytoskeleton's normal shape and structure. Abnormal tau protein hyperphosphorylation occurs in various neurodegenerative diseases, including Alzheimer's disease and certain forms of frontotemporal dementia. These findings highlight the neuroprotective potential of PSO and its constituents, suggesting their value in developing therapies for conditions characterized by tau-related abnormalities and impaired neuronal connectivity [52]. A study suggested that combining PSO with *Anredera cordifolia* extract exhibits enhanced efficacy in ameliorating age-related cognitive, thus suggesting potential for novel brain health supplements [63]. Furthermore, supplementation with PSO combined with nobiletin-rich air-dried immature ponkan powder improves cognitive function and may mitigate age-related cognitive decline in healthy elderly individuals by enhancing serum brain-derived neurotropic factor (BDNF) levels and antioxidant potential [64].

### 5.8 Anti-inflammatory activity

Perilla seed oil, rich in α-linolenic acid, reduces colitis by suppressing inflammatory markers including interleukin-1 (IL-1), interleukin-2 (IL-2), interleukin-4 (IL-4), interleukin-5 (IL-5), interleukin-6 (IL-6), and interleukin-10 (IL-10) [65,54]. Due to its antioxidant and anti-inflammatory properties, PSO is considered for therapeutic use in managing reflux esophagitis and related conditions [66]. Some studies have shown that PSO can also help with coughing and clearing mucus. Zhang et al. conducted a 90-day oral toxicity study with a 30-day recovery period to test these effects on Wistar rats [55]. The perilla seed meal may also help in managing inflammation-related conditions attributed to its high rosmarinic acid and luteolin content [8].

## 6 Recent research progresses

### 6.1 Advances in nutritional and value-addition research

Perilla seed oil holds tremendous promise as an alternative to mineral oil across various industrial applications, thanks to its multitude of health benefits. However, its high unsaturated fatty acid content presents challenges such as low thermal-oxidative stability, leading to rancidity during storage, and maintaining a liquid form over a wide temperature range [67]. Furthermore, uncertainties persist regarding the bioavailability of ω-3 fatty acids from PSO [4].

Traditionally, chemical antioxidants and physical methods have been employed to prolong the shelf life of edible oils and food products [68]. Yet, concerns over the safety of chemical antioxidants have driven research into more sophisticated methods and natural alternatives. Near-infrared spectroscopy coupled with multivariate analysis techniques has been utilized to estimate the rancidity parameters of PSO, revealing temperature as a more significant factor than humidity in peroxide generation during storage [67]. Additionally, the incorporation of supercritical fluid tomato extract has shown promise in enhancing the oxidative stability of PSO, while blending it with extra virgin olive oil improves overall stability and nutritional content [69,70].

Researchers have explored innovative methods like freeze-drying and spray-drying for microencapsulation of PSO to enhance oxidative stability [6]. Urea complexation combined with rapid preparative reversed-phase liquid chromatography has successfully isolated α-linolenic acid with high purity from PSO, while α-tocopherol has been identified as effective in reducing α-linolenic acid oxidation [56]. Supercritical carbon dioxide extraction has emerged as a superior method for obtaining PSO, offering higher antioxidant activity and storage stability compared to traditional extraction methods [71].

The bioavailability of fatty acids is highly influenced by their chemical nature, interactions with other biomolecules in food, and ion concentrations like Ca^2+^, which form complexes affecting their absorption [72]. Recent studies have specifically investigated the bioavailability of α-linolenic acid in PSO, focusing on the absorption speed and extent by the intestinal lining and entry into portal circulation. An early study using Entrox-coated PSO capsules, designed to resist fatty acid release in the stomach's acidic environment and enhance absorption in the intestine, found no significant difference in short-term α-linolenic acid pharmacokinetics compared to uncoated capsules [73]. However, a later study by Dos Santos et al. [74], designed to explore the impact of the perilla seed bran diet on ω-3 fatty acid composition in tilapia fillets, observed that the perilla seed bran diet significantly influences tilapia fillet lipid composition, with a remarkable 384 % increase in α-linolenic acid incorporation and a 74.15 % reduction in total saturated fatty acids, resulting in an improved ω-6/ω-3 fatty acid ratio.

Yogurt is a fermented product produced by lactic acid bacteria. In recent years, fortified yogurt has gained significant global attention. Zheng et al. [75] found that incorporating PSO into potato blueberry yogurt improved pH levels, water holding capacity, and antioxidative attributes. Notably, PSO interacted with lactic acid bacteria, reducing lactic acid, malic acid, and acetic acid content. This synergistic fermentation effect enhanced the yogurt's fatty acid composition, highlighting PSO as a promising fortifier for functional yogurt development.

PSO serves as a raw material for various industrial products, including paint, varnish, and ink [13]. Moreover, it contributes to the production of sustainable epoxidized vegetable oils [76] and enhances the quality of fish products like surimi. The medium- and long-chain triacylglycerols derived from α-linolenic acid in PSO are used in the deodorization process, promoting the elimination of free fatty acids and limiting the generation of *trans*-fatty acids [77]. PSO also plays a role in synthesizing biocomposite chitosan films for food preservation and eco-friendly packaging [78]. Recent innovations have demonstrated the use of microcapsules loaded with perilla seed essential oil for preserving peaches, showing its increased antibacterial and antioxidant effects [10].

Studies suggest that PSO exhibits promising potential for blending with selected oils to enhance stability and nutritional quality. Blending with palm olein, coconut oil, and groundnut oil demonstrated improved physicochemical properties, oxidative stability, and alignment with recommended fatty acid ratios, suggesting a viable strategy to elevate the functional and nutritional value of perilla oil in food applications [79]. Furthermore, exploration of customized blends could contribute to expanding the utility of perilla oil in the food industry. A recent study successfully developed a healthy blended oil, combining palm olein with PSO in two proportions (B1: 70:30, B2: 80:20 v/v). These blends exhibited superior oxidative stability (6.5 h), increased α-linolenic acid content (18 %), and achieved fatty acid ratios aligned with WHO recommendations, indicating improved thermal stability [5].

Nanoemulsion technology has shown promise in enhancing the bioavailability of PSO, with reduced particle size leading to improved absorption and antioxidant effects [80]. Inclusion complexes with γ-cyclodextrin have been explored as a means to enhance the bioavailability of perilla oil, showing promising results in both short-term and long-term studies [11,12]. Additionally, research into the lipolysis of perilla oil has highlighted the potential role of γ-cyclodextrin in improving bioavailability (Mu and Müllertz, 2015).

### 6.2 Genomics and transgenic research for enhancing oil content and quality

Genomics research in perilla has identified key genes and metabolic pathways associated with oil biosynthesis and accumulation, thus paving the way for targeted genetic modifications to enhance oil content and quality [[81], [82], [83], [84]]. Transgenic approaches have successfully introduced genes responsible for tocopherol biosynthesis and fatty acid composition modulation, resulting in transgenic perilla plants with increased nutritional value.

In a pioneering transcriptome study in perilla, Kim et al. [81] generated 392 Mb of raw sequences from four mRNA samples of seeds at different developmental stages and one mature leaf using Illumina HiSeq 2000 and assembled them into 54,079 unique transcripts, including 32,237 previously annotated genes. Among the annotated genes, 540 genes were found to be involved in known pathways of acyl-lipid metabolism. In another RNA-seq study, Zhang et al. [82] found 486 unigenes related to lipid metabolism and identified co-expressed genes and gene families regulating α-linolenic acid biosynthesis in perilla. Subsequently, Liao et al. [83] used time-course transcriptome analysis to identify genes associated with fatty acid and triacylglycerol biosynthesis in perilla seeds. In a recent study, Wu et al. [84] performed a genome-wide characterization of the *AP2/ERF* gene family, revealing its role in seed oil and α-linolenic acid synthesis in perilla. Twelve *AP2*/*ERFs* were identified as hub genes showing a significant correlation with lipid synthase genes (e.g., *FADs*, *GPAT*, and *ACSL*) and key regulatory transcription factors (e.g., *LEC2*, *IAA*, *MYB*, and *UPL3*). Furthermore, gene expression analysis identified three *AP2*/*ERFs* (*WRI*, *ABI4*, and *RAVI*) potentially playing an important role in the regulation of oil accumulation in perilla. In an effort to functionally characterize the genes for oil biosynthesis in perilla, Ohara et al. [85] introduced multiple limonene synthase gene expression constructs into tobacco to investigate enzyme activity and limonene synthesis across cellular compartments. The study demonstrated the potential for altering monoterpene synthesis in non-limonene-synthesizing hosts.

Transgenic research in perilla was initiated by the pioneering work of Kim et al. [86]. They developed a reproducible plant regeneration protocol for perilla using *Agrobacterium tumefaciens*-mediated genetic transformation. Subsequently, the γ-tocopherol methyltransferase (*γ-TMT*) gene encoding key enzymes catalyzing the final step of the tocopherol biosynthesis process, was used for trangenesis in perilla. This resulted in transgenic plants with increased α-tocopherol content, antimicrobial activity, acid value, and phenolics [87]. Sunsequently, Lee et al. [88] introduced the functional *D6DES* of the *Phytophthora citrophthora* gene under the control of the seed-specific vicilin promoter into the perilla cultivar Yeobsil. This resulted in transgenic perilla seeds accumulating significant levels of γ-linolenic acid and stearidonic acid, comprising over 45 % of the total seed oil, offering potential benefits for both human and animal health.

### 6.3 Advances in industrial applications of perilla seed oil

The industrial applications of PSO are diverse and impactful, covering pharmaceuticals, cosmetics, edible films, flavouring, animal feed, and more. Following sections and Table 6 discusses the role of PSO in industrial sector in brief.

**Table 6:** Summarized industrial application of Perilla seeds.

Source	Industrial application	Key findings	References
Perilla seed oil	Pharmaceuticals	Improved gut microbiota diversity, the promotion of beneficial bacteria, suppression of potentially harmful bacteria, and an additional source of energy	[88]
Perilla Rosmarinic acid	Cosmetic and skin care	Protective effects on human skin fibroblasts when exposed to parabens and degradation of collagen	[89]
Perilla seed oil	Cosmetic and Skin care	Anti-collagenase and anti-melanogenesis agents	[49]
Perilla aldhedye	Flavouring and seasoning	Enhances and distinguishes the taste variety of food items,	[38]
Perilla seed meals	Flavouring and seasoning	Reduce the overall fat content of the meatballs while adding the unique flavor and nutritional benefits	[90]
Perilla oil	Flavouring and seasoning	Yogurt is enriched by substituting 10 % of the ingredients with *Perilla* oil providing unique flavor profile to the yogurt	[75]

#### 6.3.1 Pharmaceutical industry

PSO plays an important role in the pharmaceutical industry as a versatile carrier for essential oils and other active ingredients. Its blending with a wide array of essential oils and pharmaceutical compounds allows for the easy creation of customized formulations. Studies reported that perilla anthocyanins can induce apoptosis in cancer cells, suggesting a potential role as an adjunct or complementary treatment in cancer therapy, particularly for cervical adenocarcinoma. In addition, incorporating PSO as a daily ω-3 fatty acid supplement yields numerous benefits, including enhanced gut microbiota diversity, promoting beneficial bacteria, suppressing potentially harmful bacteria, and providing an additional source of energy for highly active female athletes [88].

#### 6.3.2 Cosmetic and skin care

Perilla seed oil has gained significant attention for its potential to treat skin conditions, particularly atopic dermatitis (eczema). It is widely used in cosmetics and can be found in body lotions, serums, face creams, sunscreens, and dermatological preparations. It significantly contributes to the overall effectiveness of these products [91,92]. Rosmarinic acid in PSO may protect human skin fibroblasts from parabens, thus slowing down the breakdown of collagen through *pERK1/1*, *MMP1/2*, and *TIMP1/2* pathways [89]. Additionally, the residue from PSO extraction shows promise in skincare and pharmaceuticals, with anti-collagenase and anti-melanogenesis properties making it a candidate for products targeting healthy skin, addressing skin ageing, and combating hyperpigmentation [49].

#### 6.3.3 Edible films

Perilla seed meal, a by-product of perilla seed oil extraction can be used to make edible films. These films offer a sustainable and biodegradable alternative to traditional plastic packaging. When incorporated with clove oil in sausage packaging, they represent a promising approach to improving food preservation, reducing waste, and aligning with sustainability goals [93].

#### 6.3.4 Flavouring and seasoning

Perilla oil plays a significant role in enhancing the aroma and flavour of a variety of dishes, showcasing the versatility of perilla in culinary applications. Kim et al. conducted a comparative analysis of the physiochemical and sensory attributes between perilla sauces and a commercially available sauce in Korea. The commercially available sauce exhibited higher carbohydrate and sugar levels, along with higher calorie and peroxide values, in contrast to perilla oil sauces. Conversely, perilla oil sauces displayed increased crude protein and fat content. Notably, the soluble solid content in perilla oil sauces was significantly higher than that in the commercial sauce. Additionally, sauces derived from perilla oil exhibited a remarkable 12-fold increase in ω-3 polyunsaturated fatty acids and a 25 % reduction in sodium compared to the commercial sauce. Moreover, the sensory characteristics in terms of overall acceptability of perilla oil sauce were also higher than those of commercial sauce [94]. Another study by Ran et al. (2020) used perilla seed meals as a fat replacement in meatballs, presenting an opportunity to reduce overall fat content while providing the unique flavour and nutritional benefits of perilla [90]. Perilla seed oil has also been extensively used in preparing muffins and chewable tablets [95,96], and imparting a unique flavour and fatty acid profile to yogurt [75]. These culinary innovations not only expand the use of perilla but also present opportunities for enhancing both flavour and nutrition in various food products.

#### 6.3.5 Animal feed

Perilla seed meal (generally discarded after oil extraction) proves to be an invaluable source of animal feed due to the high protein content (∼30.0–40.0 %) [61]. PSM, when hydrolyzed and fractionated, exhibits potent antioxidant properties, attributed to its low molecular weight (<3 kDa) and specific amino acids such as leucine, isoleucine, proline and serine. In agreement with this, several studies suggested that perilla seed by-products, including meal and cake, can be utilized as natural antioxidants and incorporated into animal diets to improve health and enhance production [27,29,61].

## 7 Conclusion and future directions

In summary, the review highlights the nutritional, culinary, and therapeutic significance of PSO across various industries, particularly in food and pharmaceuticals. With its roots deeply embedded in the culinary heritage of Asian countries like China, Japan, and Korea, PSO not only enhances flavor but also serves as a rich source of essential nutrients, including polyunsaturated fatty acids, policosanols, phytosterols, and tocopherols. Particularly, its high concentration of ω-3 fatty acids, primarily α-linolenic acid, endows it with neuroprotective and antioxidant properties, contributing to its diverse health benefits. The extensive phytochemical content of PSO, including polyphenolic compounds further expands its therapeutic potential, ranging from anti-tumor and anti-obesity effects to cardioprotective and hepatoprotective properties. Historical consumption in Asia with minimal adverse effects highlights the nutritional advantages of properly prepared PSO, although comprehensive toxicological studies are imperative to establish safe consumption thresholds.

Future directions should focus on validating observed health benefits through clinical trials to support evidence-based health claims and pharmaceutical applications. Crop improvement strategies are vital for developing perilla varieties resilient to pests, diseases, and diverse climates, thereby enhancing yield and reliability. The exploration of climate-smart farming practices is essential to mitigate the impact of changing climate patterns on perilla cultivation. Continued genetic studies are necessary to understand the diversity of perilla, facilitating crop improvement efforts tailored to different regions. Globally promoting PSO necessitates concerted efforts in market development, product promotion, and heightened awareness of its nutritional and medicinal advantages. Addressing safety concerns through research and adopting sustainable farming practices are important steps toward exploring the full potential of PSO, making it a staple beyond its traditional Asian use.

Moving forward, future research endeavors should prioritize several key areas to further advance the understanding and utilization of PSO:

1. Clinical validation: Conducting well-designed clinical trials to validate the observed health benefits of PSO, thereby providing robust evidence for its therapeutic applications in various health conditions.
2. Crop improvement: Continuously investing in genetic studies and breeding programs to develop perilla varieties with enhanced resilience to environmental stressors, improved yield, and nutritional quality.
3. Sustainable farming practices: Exploring and implementing climate-smart farming practices to ensure the sustainable production of PSO amidst changing climate patterns, thereby safeguarding its availability for future generations.
4. Market development: Strategically promoting PSO globally through targeted marketing efforts, product diversification, and consumer education initiatives to broaden its market presence and drive demand.
5. Safety assurance: Conducting comprehensive toxicological studies to establish safe consumption thresholds and address any lingering safety concerns, thereby instilling confidence among consumers and regulatory authorities alike.

## Data availability statement

Not applicable.

## CRediT authorship contribution statement

**Simardeep Kaur:** Writing – original draft, Project administration, Investigation, Conceptualization. **Karishma Seem:** Writing – review & editing. **Ansheef Ali:** Writing – review & editing. **Sandeep Jaiswal:** Writing – review & editing. **Praveen Gumachanamardi:** Writing – review & editing. **Gurkanwal Kaur:** Writing – review & editing. **Naseeb Singh:** Writing – review & editing. **Letngam Touthang:** Writing – review & editing. **Sanjeev Kumar Singh:** Writing – review & editing. **Rakesh Bhardwaj:** Writing – review & editing, Project administration, Investigation. **Binay K. Singh:** Writing – review & editing, Project administration. **Vinay Kumar Mishra:** Writing – review & editing, Supervision. **Amritbir Riar:** Writing – review & editing, Supervision, Funding acquisition.

## Declaration of competing interest

The authors assert that they have no conflict of interest.
